# Conservation and Divergence of Regulatory Strategies at *Hox* Loci and the Origin of Tetrapod Digits

**DOI:** 10.1371/journal.pbio.1001773

**Published:** 2014-01-21

**Authors:** Joost M. Woltering, Daan Noordermeer, Marion Leleu, Denis Duboule

**Affiliations:** 1 Department of Genetics and Evolution, University of Geneva, Geneva, Switzerland; 2 School of Life Sciences, Federal Institute of Technology, Lausanne, Switzerland; New York University, United States of America

## Abstract

The evolution of tetrapod limbs from fish fins enabled the conquest of land by vertebrates and thus represents a key step in evolution. Despite the use of comparative gene expression analyses, critical aspects of this transformation remain controversial, in particular the origin of digits. *Hoxa* and *Hoxd* genes are essential for the specification of the different limb segments and their functional abrogation leads to large truncations of the appendages. Here we show that the selective transcription of mouse *Hoxa* genes in proximal and distal limbs is related to a bimodal higher order chromatin structure, similar to that reported for *Hoxd* genes, thus revealing a generic regulatory strategy implemented by both gene clusters during limb development. We found the same bimodal chromatin architecture in fish embryos, indicating that the regulatory mechanism used to pattern tetrapod limbs may predate the divergence between fish and tetrapods. However, when assessed in mice, both fish regulatory landscapes triggered transcription in proximal rather than distal limb territories, supporting an evolutionary scenario whereby digits arose as tetrapod novelties through genetic retrofitting of preexisting regulatory landscapes. We discuss the possibility to consider regulatory circuitries, rather than expression patterns, as essential parameters to define evolutionary synapomorphies.

## Abstract 2

During development, expression of the *Hoxa* and *Hoxd* genes in zebrafish fins and mouse limbs are regulated via a conserved chromatin structure. However, zebrafish lack certain regulatory elements required to produce digits, revealing that radials—the fin's bony elements—are likely not homologous to tetrapod digits.

## Author Summary

Our upper limbs differ from fish fins, notably by their subdivision into arm and hand regions, which are separated by a complex articulation, the wrist. The development of this anatomy is associated with two distinct waves of expression of the *Hoxa* and *Hoxd* genes during development. Would such a shared expression pattern be sufficient to infer homology between fish fins and mouse limbs? We investigated this question here, looking at whether the two phases of *Hox* gene transcription that are observed during tetrapod limb development also occur during zebrafish fin development. We find the answer is “not quite.” For although the mechanisms that regulate the expression of *Hoxa* and *Hoxd* are comparable between zebrafish fins and mouse limbs, when the genomic regions that regulate *Hox* gene expression in fish fins are introduced into transgenic mice, they trigger *Hox* gene expression in only the proximal limb segment (the segment nearest the body) and not in the presumptive digits. We conclude that although fish have the *Hox* regulatory toolkit to produce digits, this potential is not utilized as it is in tetrapods, and as a result we propose that fin radials—the bony elements of fins—are not homologous to tetrapod digits.

## Introduction

The tetrapod limb is made out of a proximal-to-distal series of long bones, the stylopod, zeugopod in the arm, and the digits in the hand, the latter of which are separated from the former two by the mesopodium, an articulation based on an array of small roundish bones [1]–[4]. This skeletal organisation evolved during the Devonian as an adaptation to the buoyancy-lacking environment of the land [5],[6]. The fossil record indicates that limbs evolved from fins via successive steps of distal elaboration, eventually resulting in the formation of the autopod as a tetrapod-specific evolutionary novelty, with fin radials or distal fin radials as putative evolutionary precursors of digits [7]–[9].

During mammalian limb development, the activity of both *HoxA* and *HoxD* gene clusters is essential and the absence of these two loci leads to rudimentary and truncated appendages [10]. All long bones of the limb require the activation of *Hox* genes in different though partially overlapping combinations. Initially, *Hoxd9* to *Hoxd11* and *Hoxa11* are expressed in the developing proximal limb (the presumptive arm). Subsequently, in a second phase of transcriptional activation, *Hoxd9* to *Hoxd13* as well as *Hoxa13* are expressed in presumptive digits [11]–[14]. The existence of distinct regulatory modules for long bones on either side of the mesopodial articulation (the wrist and ankle), together with the separated evolutionary trajectories of these elements, has supported the view that tetrapod limbs are genetically organized following a specific bimodal pattern of proximal (arm and forearm) and distal (digits) long bones, which as such is not present in fish fins (refs. in [1]).

**Figure 1:**
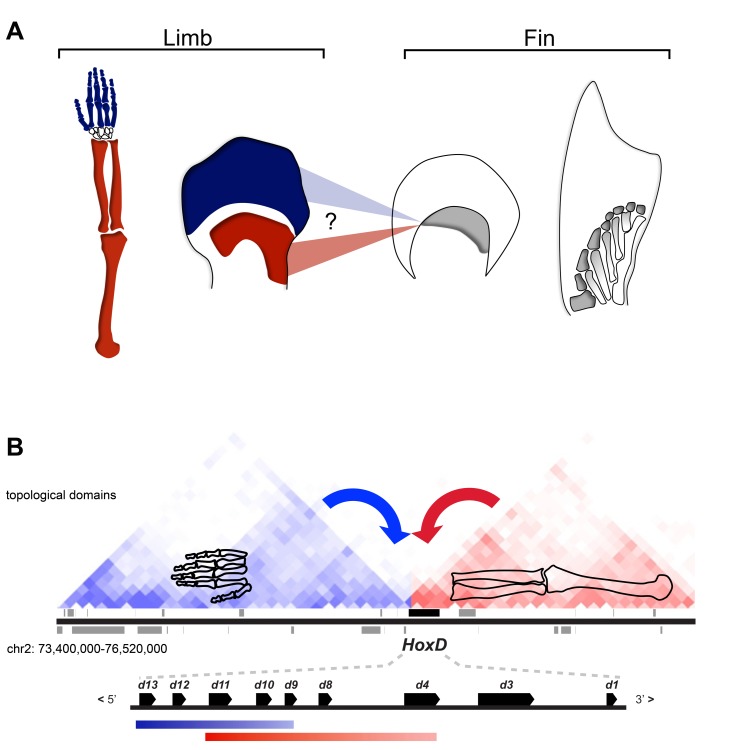
Regulatory mechanisms and the homology conundrum between fins and limbs. (A) The evolutionary changes that occurred during the transition from fins to limbs are mostly unresolved, in particular concerning the most distal segment of tetrapod limbs: the digits. Mammalian proximal and distal limb regions develop along with independent phases of *Hoxd* expression [indicated in red (arm) and blue (digits)] and fish fin buds have been probed for the existence of similar *Hoxd* expression patterns. A single expression domain of 5′ *Hoxd* genes along the distal fin margin (in grey) was interpreted either as corresponding to the distal phase in tetrapods or, alternatively, as homologous to the proximal phase [1],[15],[16],[20],[25]. Accordingly, radials (in grey) could be homologous with digits or this homology may not exist, in which case digits are tetrapod novelties. (B) Proximal (red) and distal (blue) *Hoxd* gene expression domains in the developing mouse limb are derived from enhancers located within distinct 3′- (red) and 5′- (blue) regulatory landscapes. The enhancer–promoter interaction profiles within these two landscapes were shown to precisely match two topological domains [31] as determined by Hi-C using ES cells [32]. This bimodal regulatory organization in tetrapods suggests distinct evolutionary trajectories for proximal and distal limbs. The presence or absence of such a modular regulatory strategy in fish would help clarify the origin of this mechanism and the homology between fins and limbs. The DNA domain shown is approximately 3 mb large.

The characterization of *Hoxa* and *Hoxd* expression patterns during fish fin bud development has re-enforced the view that changes in *Hox* genes' regulation were instrumental in the transformation of fins into limbs [1],[2],[15]–[19]. The exact nature of these changes, however, has remained controversial. The analysis of fin buds from various fish species lead to different conclusions regarding the existence in fishes of the late and distal phase of *Hoxd* gene expression, associated with the development of tetrapod digits (Figure 1A). This phase was indeed either considered as a tetrapod novelty [18],[20], implying an origin close to the stem of tetrapods, or alternatively as more ancestral and already present in fish [21]–[24]. This latter scenario suggests a deeper homology between fin radials and distal limb structures [16],[21],[25], which could potentially qualify distal fin radials as digit homologs [16]. Regarding *Hoxa* genes, the expression of *Hoxa11* and *Hoxa13* is largely overlapping in fins [20],[21],[24],[26], instead of the mutually exclusive patterns observed in limbs, and is thus of little help in providing a proximo-distal (P-D) reference point.

These analyses primarily rely on the comparison between gene expression domains whose interpretation is complicated when highly divergent structures are considered, such as fins and limbs [1], and hence whether or not any homology can be inferred from such expression analyses is unclear. The assessment of fish DNA sequences orthologous to tetrapod digit enhancers in transgenic mice indeed indicated their potential for regulating gene transcription in developing appendages [27],[28]. However, these fish enhancers, related to tetrapod digit control sequences, appeared to drive transgene expression primarily in more proximal mouse limb territories rather than in digits. As an alternative to using gene expression to infer homologies, we looked at whether a comparison between the regulatory mechanisms underlying *Hox* gene transcription in both tetrapod and fish appendages could be more informative.

The transcriptional regulation of the *HoxD* gene cluster during limb development becomes rather well understood. The successive proximal and distal waves of expression are controlled by distinct enhancer-containing regulatory landscapes, located in gene deserts on opposite sides of the gene cluster. A proximal landscape is located on the 3′ side of the gene cluster, whereas the distal (digit) landscape extends on the 5′ side [29]–[31]. These regulatory landscapes are regions of active enhancer-promoter interactions, as defined by chromosome conformation capture (4C), and their genomic extents and properties strongly suggest that they correspond to recently defined topological domains (Figure 1B) [31]—that is, ca. 100 kb to megabase large chromatin domains, which provide a permissive environment for long-range enhancer—promoter interactions [32],[33]. *Hoxd9* to *Hoxd11*, which are located in the central part of the gene cluster, successively interact with either one of these 3′ and 5′-located regulatory landscapes depending upon which series of enhancers are active, thus switching their contacts from one landscape to the other at the time of the transition between cells with a proximal fate to cells forming the presumptive digits [31].

In contrast, genes that are situated at either extremity of the cluster always interact within their neighbor landscape and will not switch their contacts. *Hoxd13* for instance will only interact with 5′-located enhancers and, as a consequence, will be transcribed only in the distal limb territory. Therefore, a bimodal chromatin organization of the *HoxD* locus prefigures the bimodal expression of *Hoxd* genes with their proximal and/or distal specificities, leading to the tetrapod P-D limb axis [31]. The expression patterns of *Hoxa* genes suggested that this collinear property of *Hoxd* genes may also apply to their *Hoxa* paralogs and each gene cluster on its own is capable of specifying a complete limb P-D axis, as demonstrated by the full deletion of either *HoxA* or *HoxD* [10],[34],[35]. However, whether or not these functional similarities reflect a conservation of regulatory strategies remained elusive. Here, we report that this mode of regulation is globally conserved between both gene clusters, suggesting that its emergence predated the origin of tetrapods. In addition, we looked at the situation in fishes and analyzed the regulatory potential of their *Hox* clusters in the context of transgenic mice.

## Results

### *HoxA* and *HoxD* Clusters Implement Similar Global Regulatory Strategies in Limbs

**Figure 2:**
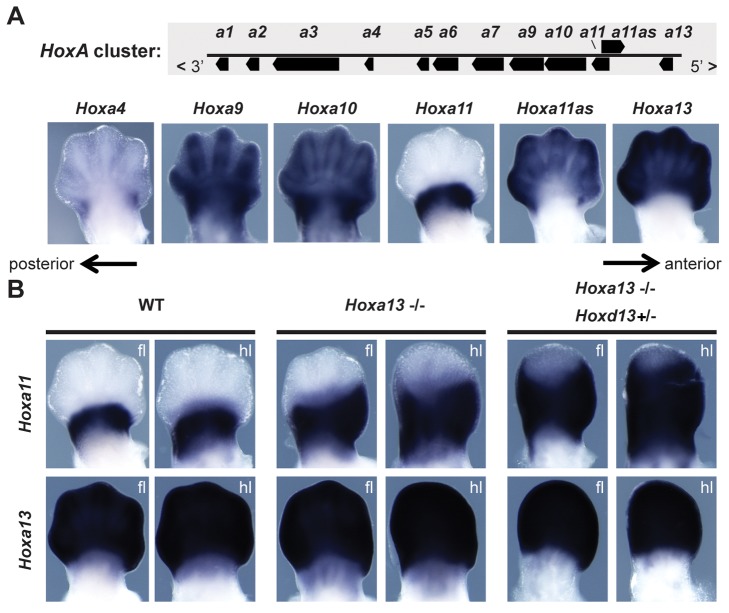
*Hoxa* gene expression in limb buds. (A) Expression of *Hoxa4*, *Hoxa9*, *Hoxa10*, *Hoxa11*, *Hoxa11* antisense (*Hoxa11as*), and *Hoxa13* in E12.5 limb buds. The *Hoxa11as* transcript [37] originates from a promoter within the intron of *Hoxa11* (upper panel) and is expressed like *Hoxa13*. (B) In control (WT) embryos, *Hoxa11* and *Hoxa13* are expressed in mutually exclusive domains, with *Hoxa13* in the autopod and *Hoxa11* in the distal zeugopod. In *Hoxa13* homozygous mutants embryos, the *Hoxa11* expression domain shifts into the proximal autopod, partly overlapping with *Hoxa13*. In *Hoxa13* ^−/−^/*Hoxd13^+/^* ^−^ double mutant animals, *Hoxa11*-expressing cells spread further distally. The *Hoxa13* probe is within the 3′ UTR and thus detects *Hoxa13* transcripts in mice carrying a loss of function for this gene. Although HOX13 proteins repress *Hoxa11* transcription, this latter gene has the capacity to respond to global distal enhancers, much like its *Hoxa9*, *Hoxa10*, *Hoxa11*, and *Hoxa13* neighbors (fl, forelimb; hl, hindlimb). The anterior-to-posterior polarity of the limb buds is indicated with arrows.

We first evaluated whether the bimodal regulatory strategy observed at the *HoxD* cluster was particular to this locus or, in contrast, was shared with *HoxA* during limb development, in which case such regulatory modalities would likely predate the duplication of *Hox* clusters and hence the emergence of tetrapods. We looked at the expression patterns of *Hoxa* genes to see how well they adhere to the proximal-to-distal restrictions observed for *Hoxd* genes in budding limbs [12],[13],[36]. Although a weak expression of *Hoxa4* was scored proximal to the digits in E12.5 limbs, strong expression of *Hoxa9* and *Hoxa10* was detected both in developing digits and in a more proximal domain, corresponding to the presumptive forearm. In contrast, both *Hoxa13* and the *Hoxa11* antisense transcript (*Hoxa11as*) [37] accumulate only in the distal, presumptive digit domain and in the future wrist (Figure 2A). A noticeable difference with *Hoxd* genes was observed, however, as *Hoxa11* transcripts are absent from this distal domain, while present in the proximal territory, suggesting that *Hoxa11* may escape the distal regulation imposed on the *Hoxa9* to *Hoxa13* genes, unlike in the case of *HoxD* [38], where *Hoxd9* to *Hoxd13* genes are coregulated in digits.

It has, however, recently been shown that *Hox13* group genes repress *Hoxa11* in the distal limb [39]. In the absence of *Hoxa13* function, the expression of *Hoxa11* shifts into the distal limb bud to partially overlap with the expression of the inactivated *Hoxa13* transcript, as detected by using a 3′UTR probe (Figure 2B, compare left and central panels). When doses of *Hoxa13* and *Hoxd13* functions were progressively removed, the distal extension was strengthened and *Hoxa11* transcripts were found almost throughout the entire developing autopod (Figure 2B, right panels), much like its antisense *Hoxa11as* transcript in the wild-type condition. Although this result indicates that both *Hoxa13* and *Hoxd13* products repress *Hoxa11* expression, it also demonstrates that the *Hoxa11* promoter can readily respond to the distal regulation, much like *Hoxa13*. Therefore, it appears that the *HoxA* cluster, like *HoxD*, is the target of a distal limb (digit) global enhancer, which can regulate at least two distinct promoters. On the other hand, *Hoxa4*, *Hoxa9*, *Hoxa10*, and *Hoxa11* are transcribed in a wider territory, including the proximal limb region, suggesting that as for the *HoxD* cluster, *Hoxa* genes are regulated by two distinct regulatory modules during limb budding and patterning.

This regulatory dichotomy may correlate with another similarity between the *HoxA* and the *HoxD* cluster—that is, the fact that *HoxA* too is located at the junction between two topological domains [32]. These data were, however, obtained in ES cells and thus we further characterized the three-dimensional chromatin dynamics of the *HoxA* cluster during limb development, in comparison with forebrain cells where all *Hox* genes are inactive, at least at this stage. We implemented circular chromosome conformation capture and deep sequencing (4C-seq) [40],[41], using *Hoxa4*, *Hoxa9*, *Hoxa11*, and *Hoxa13* as baits in E12.5 dissected presumptive digits, proximal limb, and forebrain cells. The distribution of contacts over an 8 Mb DNA interval (Figure S1A), as judged by the number of sequence reads, shows that ca. 90% of the interactions are concentrated within the regions corresponding to four topological domains as determined in ES cells and located on either side of the cluster (Figure S1B, shaded area from −2 to +2), with particularly strong contacts with the regions corresponding to the two flanking domains (Figure S1B, from −1 to +1). This observation was strongly reminiscent of the situation described for the *HoxD* cluster (Figure S1B, bottom and [31]).

**Figure 3:**
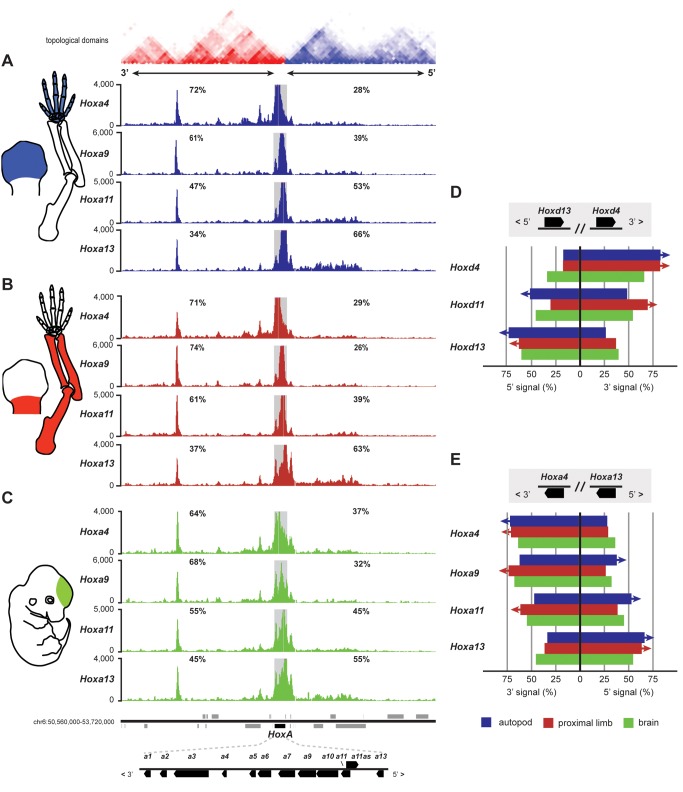
Interaction profiles of mouse *Hoxa* genes. Circular chromosome conformation capture (4C) analysis of either distal (A) or proximal (B) E12.5 dissected limb bud (schematized in the left) or forebrain (C). The proximal or distal fates of these cells are illustrated by adult skeletons (left) with the same colors as in Figure 1. Dark grey squares indicate regions of local interactions excluded from the analysis. Four interaction profiles are shown after using *Hoxa4*, *Hoxa9*, *Hoxa11*, and *Hoxa13* as viewpoints. The genomic orientation of *HoxA* is inverted with respect to *HoxD*. The percentage of contacts for each viewpoint is given, either in 5′ or in 3′ of the gene cluster. In both samples, *Hoxa4* mostly interacts with the 3′ landscape, whereas *Hoxa13* is biased toward the 5′ landscape. Both *Hoxa9* and *Hoxa11* change their bias from increased contacts in 3′, in the proximal limb bud sample (B), to contacts in 5′ in the distal sample (A), thus resembling *Hoxd* genes (Figure S2) [31]. Note that the interaction profiles obtained when using either autopod, proximal limb, or brain (C) tissues are quite similar to one another, indicating a constitutive chromatin organization at the *HoxA* locus. The size of the displayed DNA interval is of ca. 3 Mb. (D and E) Summaries of the directional 4C signals using bar diagrams in the 3′ and 5′ flanking regions of both *HoxA* and *HoxD* clusters. The colored bars represent 100% of the signal for each of the three tissues (color code at the bottom) and for three genes in either the *HoxD* (D) or the *HoxA* (E) clusters. The position of each bar with respect to the central black line (0) represents the balance between the contacts scored either in 5′ (left in D; right in E) or in the 3′ (right in D; left in E) landscapes. The *HoxA* and *HoxD* clusters are shown in opposite orientation regarding 3′ and 5′ directions to reflect their inverse locations on chromosomes 2 and 6. The four displayed topological domains were extracted from the Hi-C ES cell dataset of Dixon et al. [32].

Within the domains of high interactions (i.e., the shaded areas in Figure S1B), the occurrence of contacts was quantified and no difference was observed in the distribution of interactions for either *Hoxa4* or *Hoxa13*, when either proximal or distal limb bud samples were used. *Hoxa4* establishes interactions primarily with the 3′ neighborhood of the gene cluster in both distal (Figure 3; 72%) and proximal (71%) samples, whereas *Hoxa13* mostly contacts the opposite, 5′-located landscape in the same two samples (Figure 3; 66% and 63%, respectively) (the 3′ and 5′ orientation of the cluster is given following the direction of transcription of *Hox* genes). In contrast, both *Hoxa9* and *Hoxa11* increase their interactions with the 5′-located landscape in digit cells, when compared to proximal limb cells (Figure 3A,B; from 26% to 39% and from 39% to 53%, respectively). This shift in contacts observed with more centrally located *Hoxa* genes (*Hoxa9*, *Hoxa11*) is comparable to the situation described for the *HoxD* cluster (Figure 3D,E, Figure S2, and [29],[31]). The increase in interactions between these genes and the 5′ landscape in distal cells suggests that several *Hoxa* genes located at the 5′ extremity of the cluster are coordinately regulated in the presumptive digit domain. These results and the analogy with the *HoxD* cluster are in line with the presence of functional enhancer sequences in the 5′ landscape, capable of activating transcription with distal limb specificity (Figure S3) [42],[43].

Because the *HoxA* cluster appears to respond to a bimodal regulation that shares several features with that reported for *Hoxd* genes [31], we propose that this operational mode is a core component of both *Hoxa* and *Hoxd* gene regulation during limb proximal to distal patterning and thus likely predates the emergence of tetrapods. The data obtained from the forebrain samples show that this partitioning between 5′ and 3′ regulatory landscapes is only partially tissue-specific and largely independent from the transcriptional activity, as noted for a large proportion of topological domains (Figure 3C,D,E and Figure S2) [32],[33].

### Bimodal Partitioning of the Fish *HoxA* and *HoxD* Clusters

**Figure 4:**
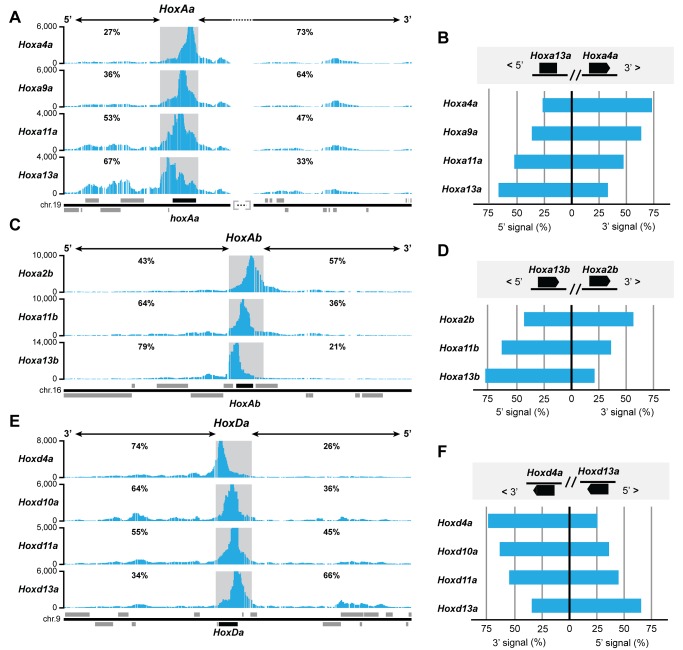
Zebrafish *Hox* clusters are partitioned into 3′ and 5′ interaction domains. 4C analysis of zebrafish whole embryos (5 dpf, including well-developed fin buds) using as viewpoints (left) several genes within the *HoxAa* (A and B), *HoxAb* (C and D), and *HoxDa* (E and F) gene clusters (for *HoxAa*, see also Figure S4). The *HoxDa* cluster has a reversed chromosomal orientation when compared to both *HoxA* clusters. The percentages of interactions between the viewpoints and either the 5′ or the 3′ landscapes are indicated above each profile. Bar diagrams in (B, D, and F) give a summary of the signal directionality per viewpoint in the 3′ and 5′ flanking regions (compare Figure 3C,D). The blue bars are as in Figure 3. Genes located at either extremity of their clusters display a strong bias toward the flanking landscape, such as *Hoxa4a* (B), *Hoxd4*a (F), *Hoxa13a* (B), or *Hoxd13a* (F). Genes located at more central positions in the clusters [e.g., *Hoxa11a* (B) or *Hoxd11a*, (F)] show more balanced interaction profiles, like for the mouse *HoxA* and *HoxD* clusters. Dark grey squares are regions of local interactions excluded from the analysis.

Because this chromatin partitioning at and around the tetrapod *HoxA and HoxD* clusters is associated with distal and proximal regulatory capacities, we looked at its presence in fish *Hox* clusters as a potential indication that distinct regulations may also be at work during fin development. Data obtained from limb tissues, brain, and ES cells all show this biased distribution in interactions corresponding to the existence of two flanking topological domains, indicating that such a structural organization exists in both expressing and nonexpressing tissues. Consequently, we used whole zebrafish embryos at day 5 postfertilization (dpf) to visualize the interaction profiles of related fish *Hox* clusters, instead of dissected fin bud tissues, which would have met our technical limitations due to their small size and the amount of tissue required for 4C analysis. Teleosts underwent an additional genome duplication and have up to eight *Hox* cluster loci [44]–[46], of which *HoxAa*, *HoxAb*, and *HoxDa* are the most relevant for fin development [24],[47]. Accordingly, we used viewpoints in *Hoxa4a*, *Hoxa9a*, *Hoxa11a*, *Hoxa13a*, *Hoxa2b*, *Hoxa11b*, *Hoxa13b*, *Hoxd4a*, *Hoxd10a*, *Hoxd11a*, and *Hoxd13a* for 4C experiments (Figure 4 and Figure S4).

We observed that fish *Hox13* genes also display a clear bias in their interactions toward their 5′ flanking neighborhood (Figure 4). For example, *Hoxa13a*, *Hoxa13b* and *Hoxd13a* show 67%, 79% and 66%, respectively, of their total contacts with their 5′ landscapes. In contrast, only 27% of the contacts established by *Hoxa4a* and 26% of the contacts established by *Hoxd4a* were scored over their 5′ landscapes, these latter genes interacting mostly with the 3′ neighborhood of the gene clusters (73% and 74% respectively). Therefore, as in the mouse, genes located at either end of the clusters establish preferential contacts with either their 3′ or 5′ neighboring landscape. In contrast, interactions involving *Hoxa9a*, *Hoxa11a*, *Hoxa11b*, *Hoxd10a*, or *Hoxd11a*—that is, genes located at more central positions—are rather equally distributed between the two landscapes on either side of the cluster (Figure 4). We thus concluded that the chromatin partitioning observed in tetrapods at the *HoxA* and *HoxD* loci is also present in fishes. These results suggest that the structural component of the mechanism underlying the distinct proximal and distal phases of *Hox* gene expression predates the evolution of tetrapod limbs. Therefore, a resemblance greater than anticipated may exist between distal fins and limb structures, as recently proposed [16],[21],[22].

### Fish Regulatory Landscapes Drive Expression in the Proximal Mouse Limb

The bias of fish *Hox13* genes to contact their immediate 5′ environment suggested that, similar to their tetrapod counterparts, they might be used as the distal contribution of a bimodal regulatory strategy. We investigated the potential presence in these fish landscapes of enhancers driving limb-specific expression. Accordingly, we generated transgenic mice with fish *Hox* clusters including their entire 5′ flanking regions. We selected Pufferfish (*Tetraodon nigroviridis*) BACs because, due to the compressed genome of this species [48], they permit the transgenic analysis of entire syntenic regions.

**Figure 5:**
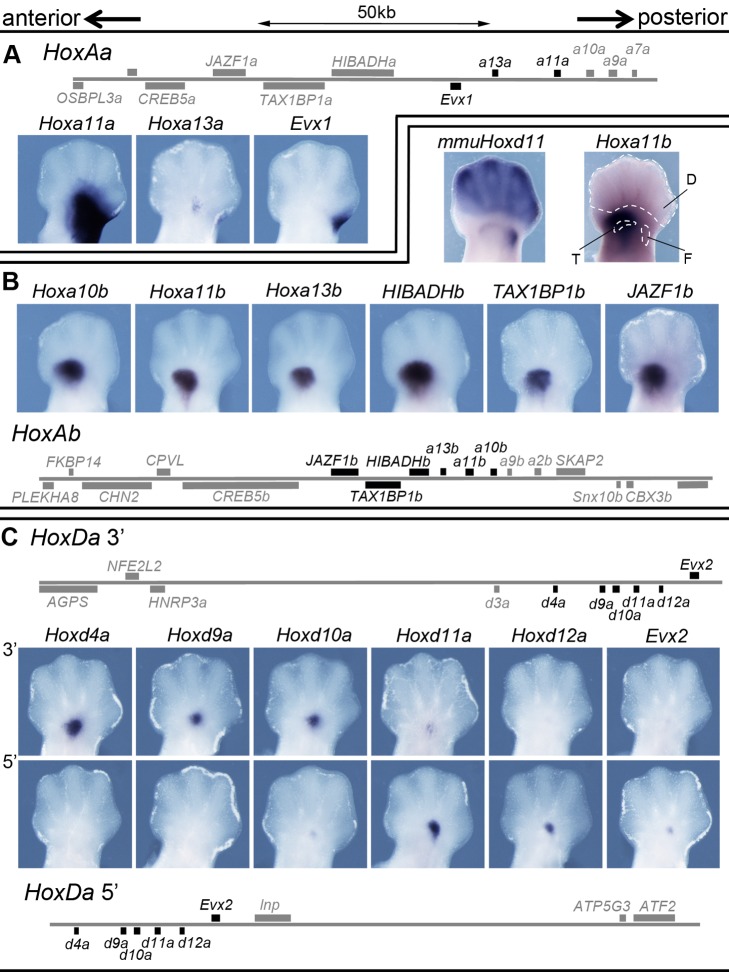
Regulatory potential of the fish *HoxA* and *HoxD* landscapes in mouse transgenic limbs. (A) Scheme of the *HoxAa* BAC used for transgenesis in the mouse with the expression of the fish *Hoxa11a*, *Hoxa13a*, and *Evx1* genes in mouse embryonic limbs. All genes assayed showed expression in a proximal domain, yet not in the presumptive digit domain. Note that *Hoxa11a* expression was not observed in forelimb buds. (B) Scheme of the *HoxAb* BAC (bottom) with the expression of several genes. The fish *Hoxa10b*, *Hoxa11b*, and *Hoxa13b* genes are expressed in a proximal domain, and transcripts are absent from the presumptive digit domain. Likewise, the 5′ flanking genes *HIBADHb*, *TAX1BP1b*, and *JAZF1b* respond to the same proximal regulation. A comparison with the endogenous *Hoxd11* expression (mmu*Hoxd11*) shows that limb expression of the transgenes is confined to the distal zeugopod and mesopod. (C) Two BAC clones containing either the entire 5′ (top) or 3′ (bottom) landscape flanking the *HoxDa* cluster with their corresponding expression patterns. Here again, expression is observed in a proximal domain but is absent from developing digits. In the various schemes, genes analyzed are shown in black. All samples are right hind limbs, dorsal views with anterior to the left, except for the endogenous mouse gene “mmu*Hoxd11*” (B), which is a mirror image of the left hind limb of the limb bud stained for *Hoxa11b* to its right, in order to facilitate the comparison of transcription domains. The anterior-to-posterior polarity is indicated with arrows. (D, digits; F and T, distal parts of the femur and tibia, respectively).

Mice transgenic for the fish *HoxAa* 5′ landscape showed expression of *Hoxa11a*, *Hoxa13a*, and *Evx1* in hindlimb buds, but with a proximal-only specificity, whereas no distal expression was observed despite the presence of the 5′ flanking genomic region (Figure 5A).

Likewise, the fish *Hoxa10b*, *Hoxa11b*, and *Hoxa13b* genes were strongly expressed in mouse limb buds transgenic for the fish *HoxAb* 5′ landscape. Here again, however, the expression domain matched a proximal zone and transcription was not scored in developing digits (Figure 5B). Because the pufferfish *HoxAb* BAC contains both the 5′ and 3′ neighborhoods, we implemented 4C-seq on transgenic mouse limbs and could confirm that strong interactions occurred between both *Hoxa13b* and *Hoxa11b* with the 5′ flanking region, despite the transcriptional outcome, which was restricted to a proximal domain (Figure S5). Also, the *HIBADHb*, *TAX1BP1b*, and *JAZF1b* genes, located next to *Hoxa13b*, were co-expressed with *HoxAb* genes, further illustrating that a global regulation is associated with this 5′ landscape (Figure 5B), as is the case for the mouse locus where these genes are co-expressed along with *Hoxa13* [49].

**Figure 6:**
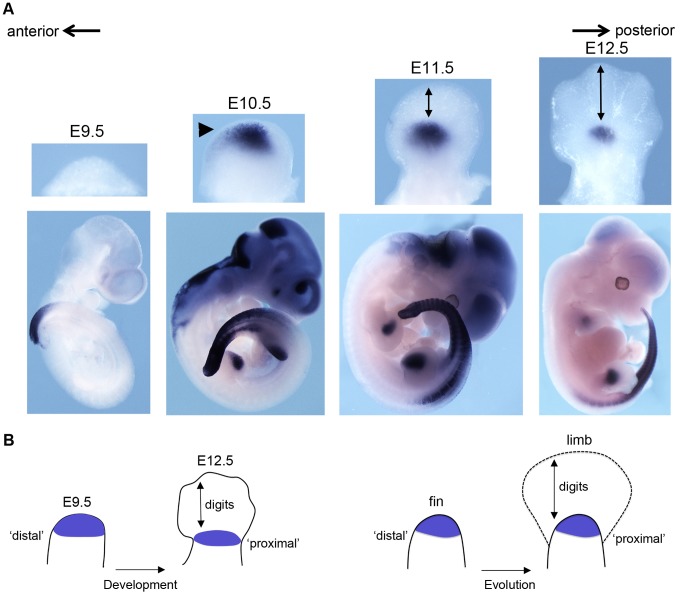
The *Tetraodon Hoxa13b* expression domain in mice: from “distal” to “proximal.” (A) *In situ* hybridization of a *Tetraodon Hoxa13b* probe using E9.5 to E12.5 fetuses transgenic for the *Tetraodon HoxAb* cluster. Top panels are dorsal views of forelimbs (anterior to the left), and bottom panels are whole mount pictures. *Hoxa13b* is expressed in limb buds and posterior trunk, whereas the staining in the head vesicles at E10.5 and E11.5 is a routinely observed artifact. At day E10.5, before the appearance of digits, expression initiates in the distal limb bud (arrowhead). In subsequent stages, however, this domain becomes increasingly “proximal” due to the distal expansion of the digit domain (arrows in E11.5 and E12.5 specimen). The distal expression of *Hoxa13b* at E10.5 is strikingly similar to the distal expression of *Hoxa13b* in the fish fin [24]. The anterior-to-posterior polarity is indicated with arrows. (B) Scheme illustrating the difficulty in using relative parameters such as “proximal” or “distal” to assign homologies. Due to the developmental expansion of the autopod, the zeugopod domain becomes relatively more proximally positioned within the limb bud, along with time. During digit evolution, a similar process may have occurred and structures that are distal in the fin apparently shifted to a more proximal position in the limb, due to the distal growth of the autopod. The fish *Hoxa13b* expression in mouse limb buds (purple color in the left scheme) in fact illustrates that distal fish fin tissues correspond to proximal limb structures after the evolution into limbs (right scheme). The fin bud scheme only depicts the endoskeletal part of the fin and not the exoskeleton, which derives from a distinct developmental lineage.

Of note, the onset of the fish *Hoxa13b* expression in transgenic limb occurs in distal limb bud cells located underneath the apical ectodermal ridge (AER; Figure 6A, arrowhead), similar to the “late” expression pattern of this gene during fin bud development. In mice, however, the expression territory of this fish transgene does not follow the distal extension of the bud and thus remains at a more proximal position (Figure 6A, arrows). This result illustrates the difficulty of using relative parameters such as “proximal” or “distal” when assigning homology between fins and limbs (Figure 6B). Altogether, the fish regions syntenic to the mouse *HoxA* cluster failed to elicit expression in presumptive digit cells during limb budding. Instead, when introduced into mice, fish *HoxA* genes were all transcribed in proximal limb domains.

We also analyzed the *HoxDa* cluster by using two BACs extending either 5′ or 3′ from the fish cluster. The 5′ BAC covers a region of the fish genome syntenic with the digit regulatory landscape, upstream of the mouse *HoxD* cluster [29], whereas the 3′ BAC is syntenic with the proximal limb regulatory landscape [31]. In both cases, when introduced into transgenic mice, the fish *Hoxda* genes were expressed in a restricted domain, always located in the proximal limb bud, whereas no transcript was detected distally (Figure 5C). In this context, the fish genes were expressed according to their relative proximity to the flanking landscapes; *Hoxd4a*, *Hoxd9a*, and *Hoxd10a* were indeed preferentially transcribed whenever their closely located 3′ landscape was included, whereas *Hoxd11a*, *Hoxd12a*, and *Evx2* were preferentially expressed when the opposite 5′ landscape was present. However, the same proximal specificity was observed in both cases, suggesting that regulatory domains exist on both sides of the fish *HoxDa* cluster, which are able to control fish *Hoxda* gene transcription in a proximal domain of the mouse limb bud, rather than in the digits (Figure 5C). These results are in agreement with the capacity of zebrafish, skate [27], and coelacanth [28] sequences orthologous to mouse *HoxD* “digit enhancers” to drive expression essentially in proximal, rather than distal, domains of murine transgenic limb buds.

## Discussion

### An Ancient Bimodal Regulatory Strategy

Our results show that, similar to the *HoxD* gene cluster, the *HoxA* cluster establishes preferential contacts with the two flanking genomic neighborhoods, such that *Hoxa13* strongly interacts with the telomeric DNA (i.e., with its 5′ side), whereas *Hoxa4* contacts its centromeric (i.e., on the 3′ side) landscape. The existence of such a structural bias in both gene clusters suggests that the ancestral gene cluster, before its duplication at the root of the vertebrate taxon, already displayed such a general bimodal chromatin structure. This may indicate the presence of a generic regulatory constraint imposed to these gene clusters, such as the necessity to functionally separate, in space and time, the most posterior genes from their anterior neighbors, the former proteins being generally dominant over the latter [50].

This split of both *HoxA* and *HoxD* clusters into two chromatin domains precisely matches the results obtained by using Hi-C on ES cell material [32]. Interestingly, however, the same dataset reveals that neither *HoxB* nor *HoxC* seem to display this feature, suggesting it may have been lost subsequently. This might relate to the fact that these latter two gene clusters are truncated either for their anterior (*HoxC*) or posterior (*HoxB*) genes.

### Constitutive Contacts

Our 4C experiments also confirmed that many interactions were present in all the tissues assayed, regardless of their transcriptional activity, as previously observed [29],[31]. In addition, the general extent of our interaction domains precisely coincided with the topological domains as defined by using the Hi-C dataset of Dixon et al. [32], further suggesting that many of those interactions associated with such topological domains are constitutive in nature. For example, the strong 3′ *HoxA* interacting peak observed at the border between topological domains −2 and −1 (Figure 3, Chr6: 51,120,000) was present in all tissues investigated. This peak colocalizes with both proximal limb enhancers (elements 406 and 407 and human element 1465) and branchial arch enhancers (elements 402 to 406), as reported in a genome-wide enhancer screen [43].

It may be that such a constitutive contact anchors the *Hox* cluster at the vicinity of tissue-specific enhancers, thus working as a priming mechanism for enhancer–promoter interactions. In this context, the presence of constitutive contacts with anchoring points rather than with the actual enhancers themselves might reflect the fact that *Hox* genes are regulated by multiple tissue-specific enhancers in time and space. The presence of a constitutive, poised regulatory architecture may have evolved at these loci to facilitate the successive implementation of multiple regulations, by providing a stable framework to be complemented by tissue-specific factors.

### The Evo-Devo-Regulo of the Fin to Limb Transition

The problem raised by the fin-to-limb transition shows that developmental expression patterns cannot always be used to infer homologies between distinct species. Because both *Hoxd13* and *Hoxa13*—that is, the two tetrapod genes essential for digit development—display this strong regulatory tropism towards their upstream genomic neighborhoods, we looked at whether the related fish *Hox* genes would display the same behavior and found that fish *Hox* gene clusters have the same conformational organization. This observation suggested a level of conservation between the regulation of these genes in both tetrapods and fishes higher than anticipated. However, the existence of such separated chromatin domains including the fish *Hox13* genes and their flanking genomic sequences does not lead to a clear partitioning of regulatory activities, at least when introduced into transgenic mice. All fish regulatory landscapes assayed, taken from either sides of the clusters, indeed elicited comparable proximal expression in mouse limbs and were thus unable to respond to those signals, triggering the emergence of the digital plate in mouse.

**Figure 7:**
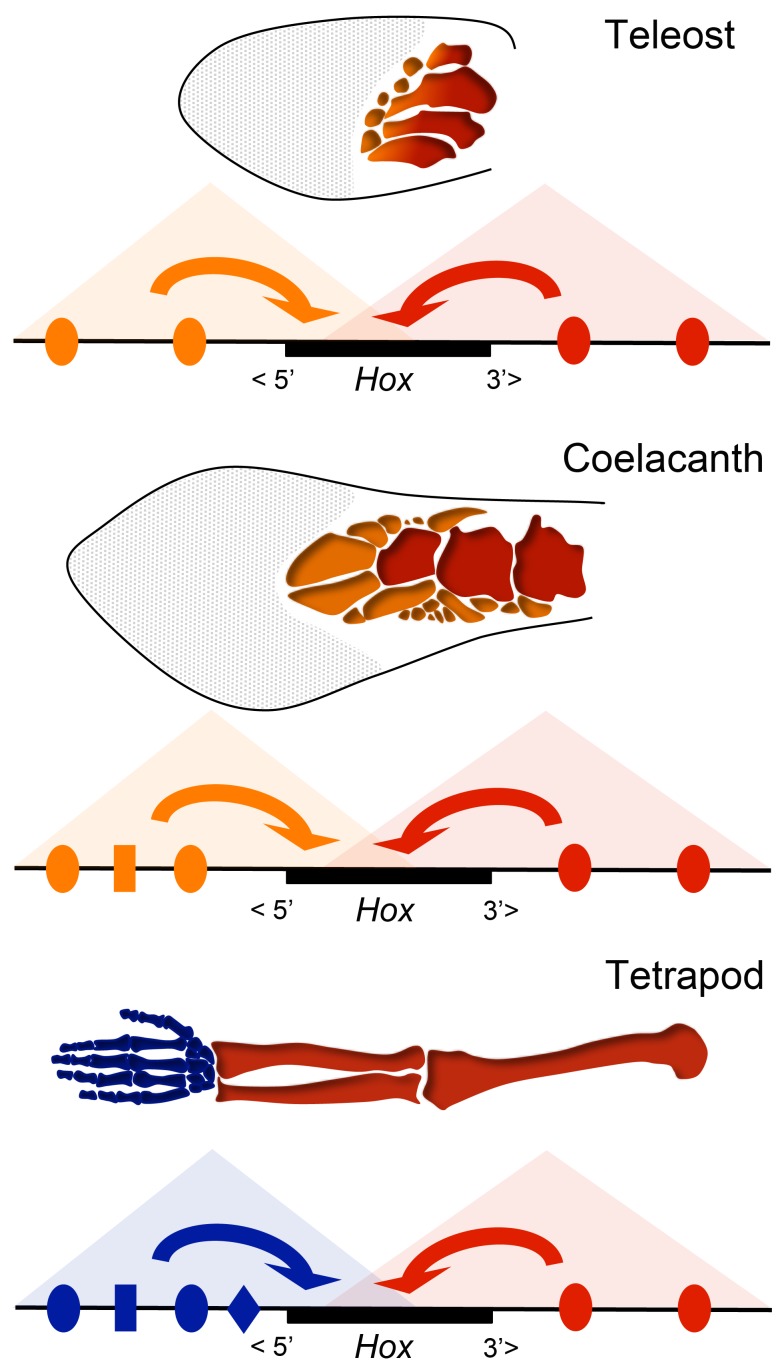
Regulatory evolution and the fin-to-limb transition. Fish and tetrapod *HoxA* and *HoxD* clusters are regulated by 3′ and 5′ regulatory landscapes, represented here as triangles due to their correspondence to topological domains [31],[32]. Enhancer (indicated with colored shapes) interactions within these domains (indicated by arrows) occur with the neighboring parts of the *Hox* clusters, resulting in a regulatory partition between 3′ and 5′ parts of the clusters. In fishes, this mechanism may be used for patterning the fin proximal (red) to distal (orange) (P-D) polarity, through the potential function of these two landscapes in slightly different fin domains. Variation in the regulatory balance between these 3′ and 5′ landscapes through the acquisition of novel enhancers potentially explains interspecies differences in P-D fin morphology, as for instance between zebrafish and species such as coelacanth, which possesses a more elaborate fin skeleton. Although these regulatory landscapes may underlie the P-D patterning of fin skeletons, they both elicit a proximal response when assessed in transgenic mice, and hence the fish 5′ landscape is indicated as “proximal” (orange). In tetrapods, the 5′ domain (blue) has acquired new enhancers or modified existing ones, thereby evolving a novel, more distal autopodial identity, perhaps as a response to preexisting signals emanating from the apical ectoderm.

The existence in fishes, of regulatory landscapes showing proximal specificities in transgenic murine limbs, may be related to the fact that fins can display elaborate proximal-to-distal patterns, as illustrated by combinations of radials and distal radials. Both zebrafish and paddlefish, as well as shark *Hox*, genes appear to be activated in a partially heterotopic manner consistent with the presence of these distinct fin segments [21],[22],[24], and hence such P-D patterns may result from biphasic regulations emanating from opposite regulatory landscapes. In this view, it is conceivable that the regulatory balance between these two landscapes in fishes contributes to the wide variety of P-D patterns observed in the fins of various species (Figure 7) [51]. Given the inability of fish regulatory sequences to activate transcription in the mouse digital plate, however, this P-D division would not be homologous to the regulatory partitioning observed between the arm and the hand in tetrapods.

### Hijacking a Regulatory Landscape

The bimodal limb *bauplan* is characterized by a clear morphological separation between the long bones of the arm and the forearm, on the one side, and of the hand, on the other side. This separation is controlled by opposite regulatory landscapes and gives rise to the presence in between of a nodular articulation critical for the function of the limbs and not observed in any fish fins: the mesopodium [1]–[3]. Because the development of long bones requires high doses of *HOX* products whereas a lower dose is associated with carpal-like small bones [52], it was proposed that the mesopodium results from the offset between the proximal and distal *Hox* expression territories, made possible by the existence of distinct regulatory landscapes [31]. In this context, a potential scenario emerges for the fin-to-limb transition whereby two partially overlapping expression domains in fins progressively segregated to generate entirely distinct expression territories.

This model hypothesizes the evolution of a bimodal “proximal–proximal” patterning system in fins (without mesopodium) into a bimodal “proximal–distal” system in limbs, including an articulation and thus postulates the transformation of a regulatory landscape from a “proximal” to a “distal” specificity. The mechanisms underlying this “regulatory homeosis” are elusive, but modifications in the structure and function of the AER, a source of growth factors in the tip of growing limb buds, may have been instrumental. During fin development, the cessation of endoskeletal expansion coincides with the transformation of the AER into the apical ectodermal fold (AEF) from which the exoskeletal fin rays will develop [53]. Various models have predicted that the folding of the ectodermal layer plays a key role in the termination of fin distal growth, possibly due to the interruption of AER-derived proliferative signals by its dense extracellular matrix [17],[18],[53]–[56]. In this view, the abrogation of ectodermal folding in tetrapods may have lead to a prolonged exposure to AER signals, leading to increased *Hox* expression and extended distal growth, thus resulting in the formation of the autopod.

However, we show here that this model cannot fully account for our transgenic results, as expression of fish *Hox* genes is not observed distal to the mesopodium, even in the absence of ectodermal folding. We conclude that there is an intrinsic inability of fish enhancers to respond to the distal limb regulatory program in the mouse. Consequently, the absence of a clear distal expression territory in fin buds separated from (but concomitant with) the proximal expression domains is likely not caused by a mere physical obstacle induced by the folding of the ectodermal layer. Rather, an ancestral fish 5′ regulatory landscape may have evolved to better respond to distal ectodermal signals, and the reinforced transcription of *Hox* genes distally might have promoted supplementary growth by delaying ectodermal folding. This situation is illustrated by the effect of overexpressing *Hoxd13a* in zebrafish fins, which leads to increased distal growth at the expense of the AEF [17]. Alternatively, the capacity for ancestral fish enhancers to respond to the appropriate distal signals may have existed from early on but be repressed, in which case tetrapod loci may have simply overcome this repression, for instance, through the loss of repressor binding sequences.

It has been pointed out that chondrichthyans or actinopterygians could be more informative regarding the fin-to-limb transition than extant teleosts [17],[21], which may have lost primitive characters. Yet fins of all these species consist of both radials and distal radials and exhibit similar *HoxA* and *HoxD* expression patterns [15],[16],[20]–[22],[24],[47]. Furthermore, our results with transgenic pufferfish sequences are consistent with the patterns found when enhancers isolated from more primitive fishes were used. In all cases indeed [27],[28], these enhancers did not elicit expression patterns as distal as one would have expected for *bona fide* digit enhancers [29],[57].

In conclusion, although digits are likely formed through the action of tetrapod-specific *Hox* enhancers, the underlying regulatory circuitry relies upon an ancestral framework already implemented in fish, illustrating the retrofitting of preexisting genomic infrastructure. In this context, the question regarding the homology between fin radials and digits may receive different answers depending on which level is considered within the regulatory hierarchy. Fish have the necessary genes and higher order regulatory architecture to form digits and likely implement the 5′ regulatory landscape to pattern distal fin radials [16]. Accordingly, digits could be considered as a specialized type of distal radials as both structures rely on a unique ancestral regulatory strategy. However, the fish 5′ regulatory landscapes are unable to specify a distinct digit territory and, as such, this regulatory feature defines a clear tetrapod synapomorphy. Therefore, a qualification of distal radials as digits (*senso* “classical” homology, that is, with a common ancestral structure) is not supported by our results.

## Materials and Methods

### Animal Experimentation and Ethics Information

All animal experiments were performed according to Swiss regulations under license no. 1008/3482/0 (to D.D.).

### Chromosome Conformation Capture

4C libraries were constructed as described before [41]. Mouse libraries consisted of 52 dissected E12.5 proximal forelimb buds, distal forelimb buds, or forebrains. Zebrafish libraries consisted of approximately 300 5 dpf embryos from the TU strain. Transgenic mouse libraries containing the *Tetraodon HoxAb* (C0AA043AG01) BAC contained 48 E12 proximal and distal hindlimb buds. The sequencing data for these samples were combined and processed as a whole limb sample. For the mouse baits used for 4C, the primary restriction enzyme used was NlaIII (New England Biolabs, R0125L), and the secondary restriction enzyme was DpnII (New England Biolabs, R0543M). For the zebrafish baits, the primary restriction enzyme was DpnII (New England Biolabs, R0543M), and the secondary enzyme was TaqαI (New England Biolabs, R0149M). In the latter case, DNA was cut for 8 h at 65°C. For the *Tetraodon* baits, assessed in transgenic mice, the primary restriction enzyme used was DpnII (New England Biolabs, R0543M), and the secondary enzyme was NlaIII (New England Biolabs, R0125L). For each viewpoint, between 1.3 and 2.6 µg of the 4C library was amplified using 16 individual PCR reactions with inverse primers containing Illumina Solexa adapter sequences (Table S2). Multiplexed samples were sequenced on the Illumina HiSeq system using 100 bp single-end reads according to the manufacturer's specifications. 4C-seq reads were sorted, aligned, and translated to restriction fragments using the 4C-seq pipeline of the BBCF HTSstation (available at http://htsstation.epfl.ch [41]). Mouse samples were mapped to the ENSEMBL Mouse assembly NCBIM37 (mm9) and zebrafish samples were mapped to the ENSEMBL Zebrafish assembly Zv9. Transgenic *Tetraodon* samples in mouse were mapped to a custom genome containing the *Tetraodon* BAC (C0AA043AG01) and ENSEMBL Mouse assembly NCBIM37 (mm9), thus minimizing the chance of mapping nonspecific reads. The directionality of signal was calculated on 4C-seq patterns over the regions mentioned in Table S1A. Data are summarized in Table S1B. In the figures, smoothed 4C-seq patterns (running mean, window size 11) are visualized except in Figure S4B, which shows unprocessed data. Topological domains shown to complement the 4C-seq experiments are ES cell HiC data take from (http://chromosome.sdsc.edu/mouse/hi-c/database.php) [32]. Mouse domains selected for 4C-seq analyses correspond to two topological domains located centromeric and telomeric of the clusters (i.e., four domains in total) and are described in Figure S1. The zebrafish regions were selected on basis of synteny with the mouse domains analyzed. Similar experiments involving the *HoxD* cluster as shown in Figure S2A,B were previously reported [31]. The experiments shown here were, however, repeated together with the analysis of *Hoxa* genes in order to compare datasets produced under the exact same conditions.

### Construction and Genotyping of Transgenic Lines

BAC constructs were identified using the Genoscope *Tetraodon* genome browser (http://www.genoscope.cns.fr/externe/tetranew/). BAC numbers and genomic positions (TETRAODON8) correspond to *HoxAa*, C0AB048AA04 (Chr21:2,888,799–3,037,908); *HoxAb*, C0AA043AG01 (Chr8:6,699,347–6,844,622); *HoxDa* 3′, C0AB015CD05 (Chr2:13,313,310–13,458,212); *HoxDa* 5′, C0AB043BH04 (Chr2:13,417,090–13,578,446). BAC clones were obtained from Genoscope, France. A PISceI meganuclease site was introduced into the vector backbones using standard EL250 cell-based recombineering technology. BAC DNA was prepared using a Nucleobond Midiprep Kit, linearized with PISceI (New England Biolabs, R0696L), incubated with SDS according to the manufacturer's instructions, 2× chloroform-phenol purified, ethanol precipitated, and dialyzed against microinjection buffer containing protamines [70 µM spermidine (Sigma, S2626), 30 µM spermine (Sigma, S3256)]. Constructs were microinjected using standard protocols for pronuclear injection. BAC lines were genotyped using primer pairs every 5 to 10 kb in combination with deep sequencing using 4C-seq and mapping of the reads on the BAC sequence to confirm its integrity. A *HoxAb* transgenic line was mapped using embryonic hindlimb samples (Figure S5), and adult mouse ear samples of *HoxDa* 3′ and 5′ BAC lines were processed using 4C-seq specifically for the purpose of integrity mapping using a viewpoint located in *Hoxd11a* (unpublished data). BAC diagrams in Figure 5 represent the regions that were found to be integrated using PCR (all lines) or 4C-seq data (*HoxAb*, *HoxDa* 3′, and *HoxDa* 5′) in the transgenic lines presented.

### Mutant Mouse Lines and *in Situ* Hybridization

The *Hoxa13* and *Hoxd13* mouse knockout lines were previously described [58],[59]. *In situ* hybridization was performed as described [60] with a temperature of prehybridization, hybridization, and posthybridization steps increased to 68.5°C. For *Tetraodon* probes, the SSC concentration in the hybridization mix was lowered to 1.3× to increase specificity (for the *Evx2* probe, 0.5×SSC was used), and the posthybridization SSC washes were done using 4×30 minutes 2×SSC-T. In all experiments using transgenic *Tetraodon* probes, wild-type embryos were coprocessed for each probe and stage to monitor specificity of the probes (unpublished data). Except in brain vesicles, susceptible to probe trapping, nonspecific signal was never observed using the conditions described above.

### Probe Construction

Probes were amplified using PCR from BAC DNA or limb cDNA and cloned into pGEMTE easy vector systems (Promega A1360). Primer sequences are given in Table S3. DIG-labeled RNA probes were synthesized using Sp6 or T7 polymerase (Promega). Probes for *Hoxd11* and *Hoxa13* were described previously [61],[62]. The probe used to detect the *Hoxa11* sense transcript was kindly provided by Dr. C. Fromental-Ramain and corresponds to a ScaI-HpaI fragment in the 3′ UTR of *Hoxa11* (mm10: Chr6:52,242,847–52,243,385).

### Image Acquisition and Editing

*In situ* hybridization images were acquired using Leica Application Suite software v3.3.1 in combination with a Leica DFC300FX camera and Leica MZFLIII microscope. Images were edited in Adobe Photoshop software using the brightness/contrast function. Limb buds shown in Figure 1B stained for *Hoxa13* are right side limb buds coming from the same embryos as the left side limb buds stained for *Hoxa11* and are mirrored for purpose of comparison. Similarly, the *Hoxa4* sample in Figure 1A is a mirror image right side forelimb bud. In Figure 1A the same wild-type forelimb specimen is used as in Figure 1B to illustrate the wild-type expression of *Hoxa11*.

### Accession Numbers

Unprocessed 4C-seq data for mouse and zebrafish samples are available from the Gene Expression Omnibus repository under accession number GSE47644. Ensembl IDs for genes used in this study are as follows: Mouse (*Mus musculus*), *Hoxa4*; ENSMUSG00000000942| *Hoxa9*; ENSMUSG00000038227| *Hoxa10*; ENSMUSG00000000938| *Hoxa11*; ENSMUSG00000038210| *Hoxa11as*; ENSMUSG00000086427| *Hoxa13*; ENSMUSG00000038203| *Hoxd4*; ENSMUST00000111980| *Hoxd11*; ENSMUSG00000042499| *Hoxd13*; ENSMUSG00000001819|; Zebrafish (*Danio rerio*), *Hoxa4a*; ENSDARG00000057724| *Hoxa9a*; ENSDARG00000096510| *Hoxa11a*; ENSDARG00000009045| *Hoxa13a*; ENSDARG00000007609| *Hoxa2b*; ENSDARG00000023031| *Hoxa9b*; ENSDARG00000007009| *Hoxa13b*; ENSDARG00000036254| *Hoxd4a*; ENSDARG00000059276| *Hoxd10a*; ENSDARG00000057859| *Hoxd11a*; ENSDARG00000059267| *Hoxd13a*; ENSDARG00000059256|; Pufferfish (*Tetraodon nigroviridis*), *Hoxa11a*; ENSTNIG00000001767| *Hoxa13a*; ENSTNIG00000009207| *Evx1*; ENSTNIG00000000875| *Hoxa10b*; ENSTNIG00000001780| *Hoxa11b*; ENSTNIG00000000494| *Hoxa13b*; ENSTNIG00000001781| *HIBADHb*; ENSTNIG00000018428| *TAX1BP1b*; ENSTNIG00000018429| *JAZF1b*; ENSTNIG00000018430| *Hoxd4a*; ENSTNIG00000001765| *Hoxd9a*; ENSTNIG00000016957| *Hoxd10a*; ENSTNIG00000001775| *Hoxd11a*; ENSTNIG00000001776| *Hoxd12a*; ENSTNIG00000001777| *Evx2*; ENSTNIG00000001817|.

## Supporting Information

Figure S1 **Interaction profiles around the mouse** ***HoxA*** **and** ***HoxD*** **genomic loci.** (A) Distribution of 4C signals (in percent of total interactions) over large genomic intervals flanking the *HoxA* and *HoxD* clusters, including 10 topological domains (−5 to +5 from centromeric to telomeric, respectively, with domains −1 and +1 neighboring the clusters) as determined by using the Hi-C dataset of [32]. The genomic coordinates for these regions are as follows: *HoxA*, Chr6:47,160,000–55,960,000 and *HoxD*, Chr2:70,960,000–80,160,000. The 4C signals are combined for all viewpoints after normalization, either in the *HoxA* or in the *HoxD* cluster (i.e., *Hoxa4*, *Hoxa9*, *Hoxa11*, *Hoxa13 Hoxd4*, *Hoxd11*, and *Hoxd13*). For each cluster, the diagram on the left shows the percentage of reads localized within each topological domain (indicated in percent), whereas the diagram on the right shows the percentage of reads per megabase (indicated as %/Mb) within each topological domains—that is, after correction for the size of the domains. The legend to the color code referring to the topological domains (numbering after [32]) is on the right. A vast majority of contacts are established within the DNA interval covered by the first two topological domains flanking the *Hox* clusters on either side (i.e., −1, −2, +1, +2), demonstrating the correspondence between the 4C data and the organization into topological domains [32]. DNA regions of strong local interactions [41] directly surrounding the viewpoints were excluded from the analysis. For *HoxA*, these are from Chr6:52,098,978–52,227,163, and for *HoxD*, from Chr2:74,484,971–74,607,492. (B) Interaction profiles for the *HoxA* and *HoxD* cluster in dissected digit (autopod) samples using viewpoints located in *Hoxa4* and *Hoxa13* (top) or *Hoxd4* and *Hoxd13* (bottom). A DNA interval containing six topological domains is shown (Hi-C heatmap data from [32]). For *Hoxa4* (top), robust centromeric (3′) interactions are scored up to the boundary between topological domains −2 and −3 (in red), whereas on the telomeric (5′) side, *Hoxa13* mostly interacts with domain +1 and weaker contacts are detected in domain +2 (blue). The same interaction patterns are seen for *Hoxd* genes, with clear thresholds in contacts occurring either at the boundary between topological domain −2 and −3 in the 5′ contacts (blue) or in 3′ between topological domains +2a and +2b (below and [31]). Consequently, these four topological domains (two on each side, shadowed DNA intervals) were selected for further analyses as presented in Figure 3.
(TIF)

Figure S2 **Interaction profiles of murine** ***HoxD*** **cluster genes.** (A–C) 4C analysis using *Hoxd4*, *Hoxd11*, and *Hoxd13* as viewpoints, in either E12.5 proximal limbs, autopod, or forebrain tissues. (A and B) In the limbs, *Hoxd4* and *Hoxd13* show strong interaction preferences for the topological domains located 3′ and 5′ of the cluster, respectively, whereas *Hoxd11* switches from 3′ to 5′ enriched contacts between the proximal limb and the autopod samples (see [31]). (C) The 3′ and 5′ bias in contact distribution for *Hoxd4* and *Hoxd13* is also present in inactive forebrain cells.
(TIF)

Figure S3 **Limb enhancers in regions flanking the** ***HoxA*** **cluster.** Limb enhancers with proximal and distal specificities have been reported in regions flanking the *HoxA* cluster. The drawings illustrate published datasets (see below). Vista mm407 (A) and Vista hs1465 (B) are located 3′ from the cluster and drive expression in proximal areas of the limb bud. In the 5′ region, enhancer activity recapitulated part of the *Hoxa13* expression pattern in the autopod. Vista mm48 (C) drives expression in the distal hind limb bud, whereas Vista hs1430 (E) shows both proximal and distal specificities. The Vista enhancer sequences are after [43] (http://enhancer.lbl.gov/frnt_page_n.shtml). The regulatory activity of BAC RPCI-23-347D13 (D), which recapitulates digit expression in a *Hoxa13*-like pattern, is after [42].
(TIF)

Figure S4 **4C analysis of** ***HoxAa*** **in Zebrafish embryos.** (A and B) For all the viewpoints located in the fish *HoxAa* cluster, the distribution of 4C signals displayed a surprising gap in 3′ located regions, with an abrupt drop around the start of *ube2e1* and a sudden recovery ca. 750 kb more 3′, at the start of *KANSL1* (unsmoothed raw signal shown in B). These abrupt transitions are unusual in 4C interaction patterns, which typically show a smooth gradient between sequences located in each other's proximity as well as a higher “background” signal between regions *in cis* showing strong interactions, and hence we considered the possibility of an error in the current assembly of the zebrafish genome. The zebrafish genome is assembled based on whole genome shotgun sequencing (WGS, indicated in red) and a clone path of overlapping BAC clones (“Ctg,” in green), of which the latter is of higher quality (http://www.sanger.ac.uk/Projects/D_rerio/faqs.shtml). The region surrounding the *HoxAa* cluster consists of three contigs (Ctg1861, Ctg1872, Ctg1864). Clone contigs are in general well assembled within themselves, but their position relative to each other is not always certain. The observed signal drop corresponds to Ctg1872 plus its 5′ flanking region of whole genome shotgun assembly, up to the start of *ube2e1*. This region contains genes that do not belong to the synteny 3′ of the *HoxA* cluster, a region otherwise well conserved in other vertebrates. Ctg1872 was mapped to its current position using a genetic map, which however is rather uninformative at this close proximity to the centromere, and this contig could equally be well positioned somewhere on the 5′ side of Ctg1861—that is, much further away from the *HoxAa* cluster (James Torrance, Sanger Institute, Zebrafish genome project, personal communication). Ctg1864 appears reliably placed, with a synteny in line with the vertebrate conservation profile (*npvf*, *cycsa*, *osbpl3a*). In the absence of Ctg1872, the region of WGS assembly 3′ of Ctg1864 (containing *ColQ*, *Snx10a*, *Cbx3a*, and *Nfe2l3*) would form an uninterrupted gene synteny between Ctg1861 and Ctg1864, and hence we conclude that the placement of Ctg1872 is most likely incorrect and we thus excluded it from the data presented (dotted line Figure 4A). In any case, the analysis including or excluding Ctg1872 plus 5′ flanking WGS only leads to a marginal difference (increased 3′ bias in interaction of <4%) and does not change our interpretation or conclusions.
(TIF)

Figure S5 **4C interaction profiles from the transgenic** ***Tetraodon HoxAb*** **cluster.** 4C-seq signals obtained from E12.5 mouse limb buds transgenic for the *Tetraodon HoxAb* cluster, using *Hoxa11b* and *Hoxa13b* as viewpoints (positions indicated with red asterisks). The 4C-seq profiles show strong interactions between the fish genes (viewpoints) and the 5′ BAC region. In addition, the presence of a smooth signal curve over the entire length of the BAC demonstrates its integrity at the integration site of the transgenic mouse line.
(TIF)

Table S1 **Directionality of 4C interactions on both 5′ and 3′ sides of mouse and fish** ***Hox*** **cluster.**
(PDF)

Table S2 **List of primers used for 4C-seq analyses.**
(PDF)

Table S3 **List of primers used to clone DNA probe for whole mount** ***in situ*** **hybridizations.**
(PDF)

## Abbreviations

4C: Circularized Chromatin Conformation Capture
AEF: apical ectodermal fold
AER: apical ectodermal ridge
BAC: bacterial artificial chromosome
Chr: chromosome
dpf: days postfertilization
kb: kilobase
Mb: megabase
mmu: *Mus musculus*
P-D: proximo-distal
WT: wild-type
