# Supplementary figures and images for: Conservation and Divergence of Regulatory Strategies at Hox Loci and the Origin of Tetrapod Digits
Source: PLoS Biol. 2014 Jan 21;12(1):e1001773. doi: 10.1371/journal.pbio.1001773 (PMC3897358; doi:10.1371/journal.pbio.1001773)

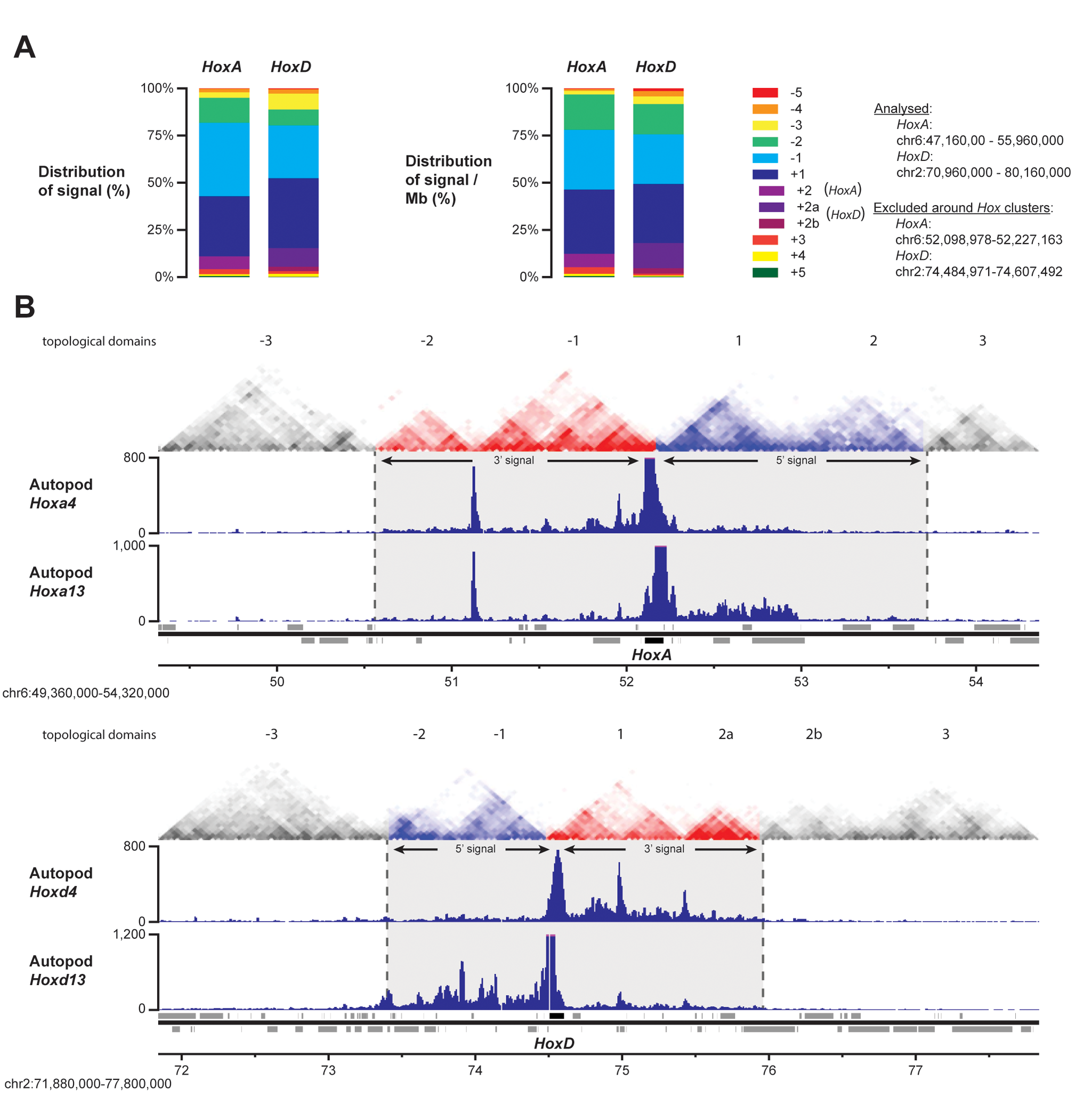

Supplement: Figure S1 — Interaction profiles around the mouse HoxA and HoxD genomic loci. (A) Distribution of 4C signals (in percent of total interactions) over large genomic intervals flanking the HoxA and HoxD clusters, including 10 topological domains (−5 to +5 from centromeric to telomeric, respectively, with domains −1 and +1 neighboring the clusters) as determined by using the Hi-C dataset of [32]. The genomic coordinates for these regions are as follows: HoxA, Chr6:47,160,000–55,960,000 and HoxD, Chr2:70,960,000–80,160,000. The 4C signals are combined for all viewpoints after normalization, either in the HoxA or in the HoxD cluster (i.e., Hoxa4, Hoxa9, Hoxa11, Hoxa13 Hoxd4, Hoxd11, and Hoxd13). For each cluster, the diagram on the left shows the percentage of reads localized within each topological domain (indicated in percent), whereas the diagram on the right shows the percentage of reads per megabase (indicated as %/Mb) within each topological domains—that is, after correction for the size of the domains. The legend to the color code referring to the topological domains (numbering after [32]) is on the right. A vast majority of contacts are established within the DNA interval covered by the first two topological domains flanking the Hox clusters on either side (i.e., −1, −2, +1, +2), demonstrating the correspondence between the 4C data and the organization into topological domains [32]. DNA regions of strong local interactions [41] directly surrounding the viewpoints were excluded from the analysis. For HoxA, these are from Chr6:52,098,978–52,227,163, and for HoxD, from Chr2:74,484,971–74,607,492. (B) Interaction profiles for the HoxA and HoxD cluster in dissected digit (autopod) samples using viewpoints located in Hoxa4 and Hoxa13 (top) or Hoxd4 and Hoxd13 (bottom). A DNA interval containing six topological domains is shown (Hi-C heatmap data from [32]). For Hoxa4 (top), robust centromeric (3′) interactions are scored up to the boundary between topological domains −2 and −3 (in [file pbio.1001773.s001.tif]

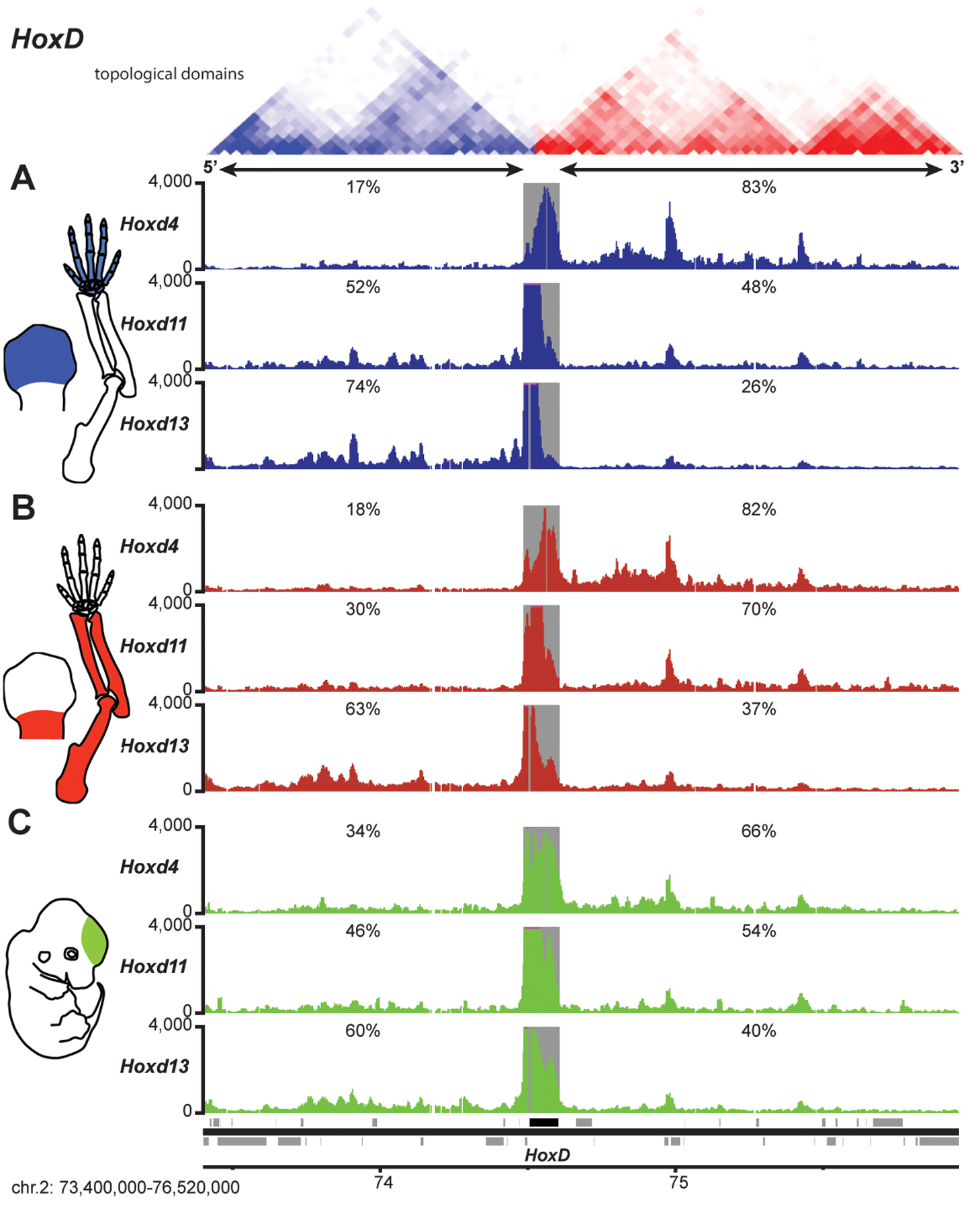

Supplement: Figure S2 — Interaction profiles of murine HoxD cluster genes. (A–C) 4C analysis using Hoxd4, Hoxd11, and Hoxd13 as viewpoints, in either E12.5 proximal limbs, autopod, or forebrain tissues. (A and B) In the limbs, Hoxd4 and Hoxd13 show strong interaction preferences for the topological domains located 3′ and 5′ of the cluster, respectively, whereas Hoxd11 switches from 3′ to 5′ enriched contacts between the proximal limb and the autopod samples (see [31]). (C) The 3′ and 5′ bias in contact distribution for Hoxd4 and Hoxd13 is also present in inactive forebrain cells. (TIF) [file pbio.1001773.s002.tif]

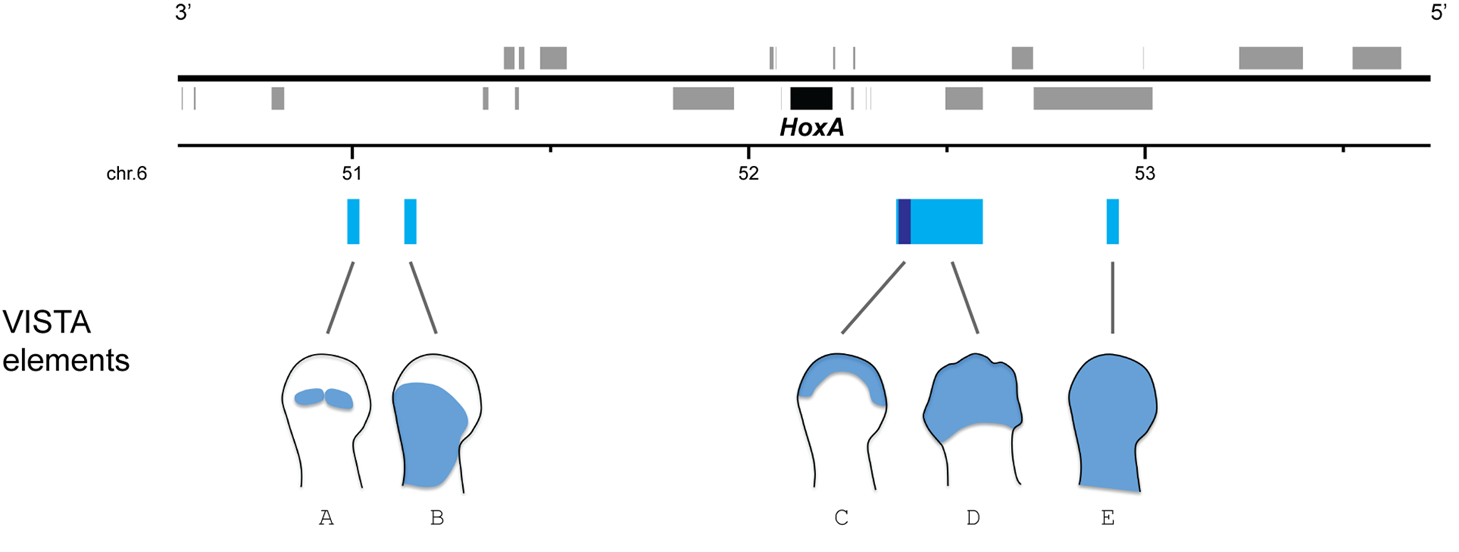

Supplement: Figure S3 — Limb enhancers in regions flanking the HoxA cluster. Limb enhancers with proximal and distal specificities have been reported in regions flanking the HoxA cluster. The drawings illustrate published datasets (see below). Vista mm407 (A) and Vista hs1465 (B) are located 3′ from the cluster and drive expression in proximal areas of the limb bud. In the 5′ region, enhancer activity recapitulated part of the Hoxa13 expression pattern in the autopod. Vista mm48 (C) drives expression in the distal hind limb bud, whereas Vista hs1430 (E) shows both proximal and distal specificities. The Vista enhancer sequences are after [43] (http://enhancer.lbl.gov/frnt_page_n.shtml). The regulatory activity of BAC RPCI-23-347D13 (D), which recapitulates digit expression in a Hoxa13-like pattern, is after [42]. (TIF) [file pbio.1001773.s003.tif]

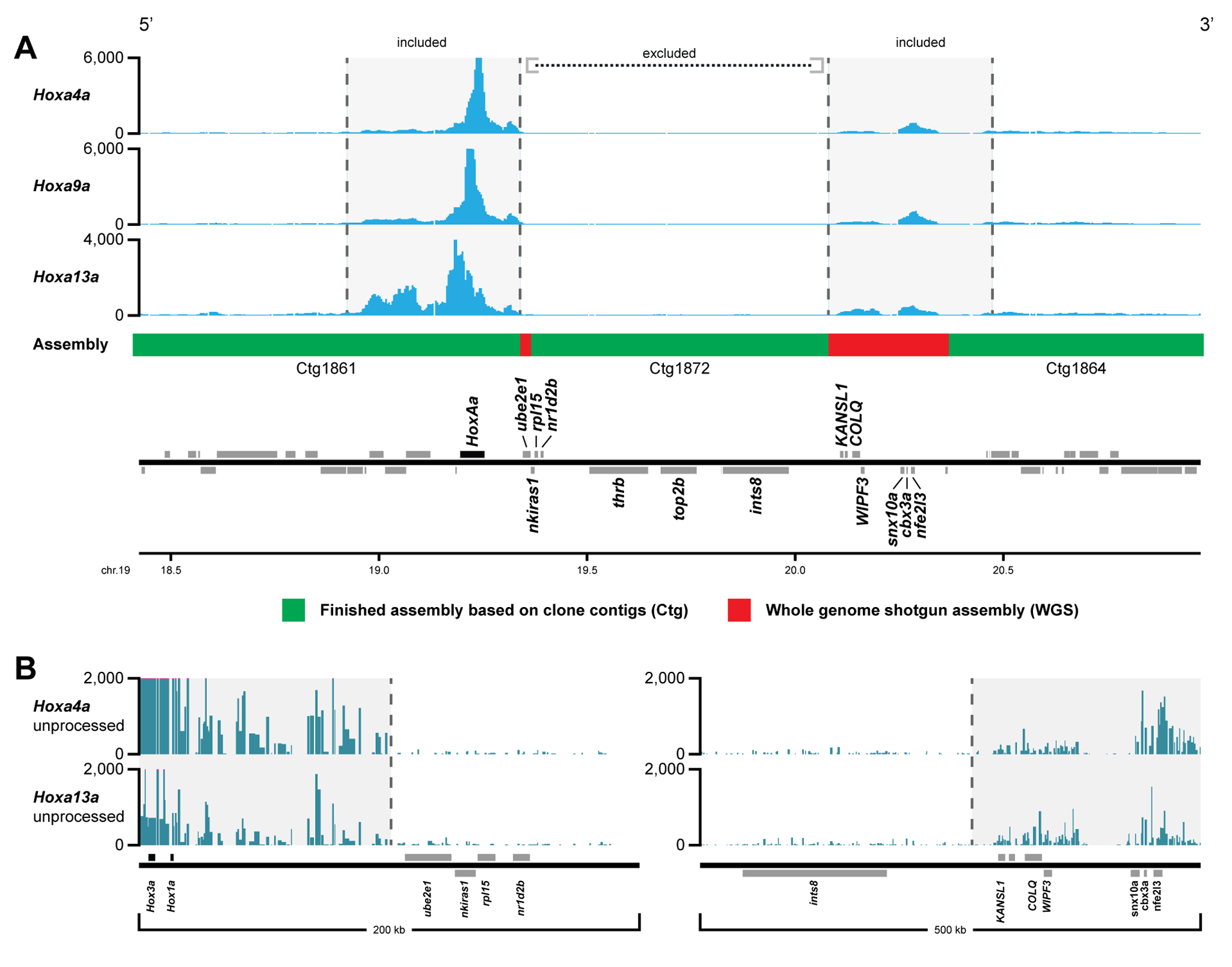

Supplement: Figure S4 — 4C analysis of HoxAa in Zebrafish embryos. (A and B) For all the viewpoints located in the fish HoxAa cluster, the distribution of 4C signals displayed a surprising gap in 3′ located regions, with an abrupt drop around the start of ube2e1 and a sudden recovery ca. 750 kb more 3′, at the start of KANSL1 (unsmoothed raw signal shown in B). These abrupt transitions are unusual in 4C interaction patterns, which typically show a smooth gradient between sequences located in each other's proximity as well as a higher “background” signal between regions in cis showing strong interactions, and hence we considered the possibility of an error in the current assembly of the zebrafish genome. The zebrafish genome is assembled based on whole genome shotgun sequencing (WGS, indicated in red) and a clone path of overlapping BAC clones (“Ctg,” in green), of which the latter is of higher quality (http://www.sanger.ac.uk/Projects/D_rerio/faqs.shtml). The region surrounding the HoxAa cluster consists of three contigs (Ctg1861, Ctg1872, Ctg1864). Clone contigs are in general well assembled within themselves, but their position relative to each other is not always certain. The observed signal drop corresponds to Ctg1872 plus its 5′ flanking region of whole genome shotgun assembly, up to the start of ube2e1. This region contains genes that do not belong to the synteny 3′ of the HoxA cluster, a region otherwise well conserved in other vertebrates. Ctg1872 was mapped to its current position using a genetic map, which however is rather uninformative at this close proximity to the centromere, and this contig could equally be well positioned somewhere on the 5′ side of Ctg1861—that is, much further away from the HoxAa cluster (James Torrance, Sanger Institute, Zebrafish genome project, personal communication). Ctg1864 appears reliably placed, with a synteny in line with the vertebrate conservation profile (npvf, cycsa, osbpl3a). In the absence of Ctg1872, the region of WGS assembly 3′ of Ctg18 [file pbio.1001773.s004.tif]

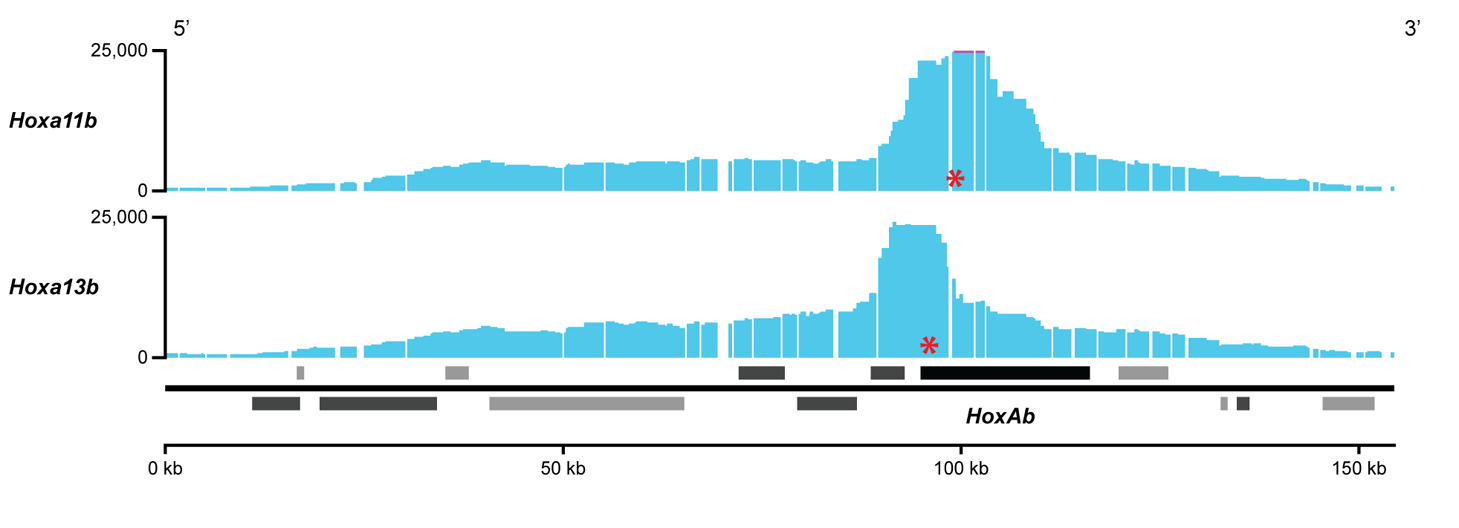

Supplement: Figure S5 — 4C interaction profiles from the transgenic Tetraodon HoxAb cluster. 4C-seq signals obtained from E12.5 mouse limb buds transgenic for the Tetraodon HoxAb cluster, using Hoxa11b and Hoxa13b as viewpoints (positions indicated with red asterisks). The 4C-seq profiles show strong interactions between the fish genes (viewpoints) and the 5′ BAC region. In addition, the presence of a smooth signal curve over the entire length of the BAC demonstrates its integrity at the integration site of the transgenic mouse line. (TIF) [file pbio.1001773.s005.tif]
