# Supplementary material for: Conservation and Divergence of Regulatory Strategies at Hox Loci and the Origin of Tetrapod Digits
Source: PLoS Biol. 2014 Jan 21;12(1):e1001773. doi: 10.1371/journal.pbio.1001773 (PMC3897358; doi:10.1371/journal.pbio.1001773)
Supplement: Table S1 — Directionality of 4C interactions on both 5′ and 3′ sides of mouse and fish Hox cluster. (PDF) [file pbio.1001773.s006.pdf]

Table S1: Directionality of 4C-seq on 5′ and 3′ sides

A. Analysed region

Mouse

	Analysed region	Excluded region around *Hox* cluster
HoxA	chr6:50,560,000-53,720,000	chr6:52,098,978-52,227,163
HoxD	chr2:73,400,000-75,960,000	chr2:74,484,971-74,607,492

Zebrafish

	Analysed region	Excluded region around *Hox* cluster
HoxAa	chr19:18,923,398-20,473,964*	chr19:19,163,605-19,259,166
HoxAb	chr16:22,639,378-23,326,921	chr16:22,965,776-23,034,186
HoxDa	chr9:1,487,878-2,469,878	chr9:1,923,143-1,996,717

* additionally, the incorrectly mapped region chr19:19,339,227-20,079,754 has been excluded see **Figure S4**.

B. Distribution of signal in 5′ and 3′ sides

Mouse

	Autopod		Zeugopod		Brain	
	3′ signal	5′ signal	3′ signal	5′ signal	3′ signal	5′ signal
Hoxa4	469,563 (72.1%)	181,849 (27.9%)	461,734 (71.1%)	187,457 (28.9%)	418,224 (63.8%)	236,881 (36.2%)
Hoxa9	341,659 (62.3%)	207,184 (37.7%)	430,871 (73.6%)	154,556 (26.4%)	432,454 (67.6%)	206,802 (32.4%)
Hoxa11	277,989 (47.2%)	311,319 (52.8%)	366,920 (61.4%)	230,237 (38.6%)	352,240 (54.8%)	290,762 (45.2%)
Hoxa13	201,363 (33.7%)	396,172 (66.3%)	258,587 (36.6%)	448,876 (63.4%)	304,885 (45.5%)	365,571 (54.5%)

	Autopod		Zeugopod		Brain	
	5′ signal	3′ signal	5′ signal	3′ signal	5′ signal	3′ signal
Hoxd4	130,255 (17.3%)	622,657 (82.7%)	134,289 (17.6%)	628,719 (82.4%)	218,754 (34.0%)	424,405 (66.0%)
Hoxd11	274,863 (51.6%)	257,492 (48.4%)	183,077 (30.4%)	418,402 (69.6%)	250,553 (45.7%)	297,563 (54.3%)
Hoxd13	432,852 (73.5%)	155,735 (26.5%)	436,234 (63.1%)	255,335 (36.9%)	367,937 (60.4%)	240,900 (39.6%)

Zebrafish

	hoxa4a		*hoxa9a*		*Hoxa11a*		*hoxa13a*	
	5′ signal	3′ signal	5′ signal	3′ signal	5′ signal	3′ signal	5′ signal	3′ signal
HoxAa	25,468 (26.6%)	70,220 (73.4%)	40,382 (36.4%)	70,452 (63.6%)	51,685 (49.6%)	52,557 (50.4%)	95,967 (66.9%)	47,455 (33.1%)

	hoxa2b		*hoxa11b*		*hoxa13b*	
	5′ signal	3′ signal	5′ signal	3′ signal	5′ signal	3′ signal
HoxAb	46,237 (43.4%)	60,381 (56.6%)	73,275 (63.8%)	41,553 (36.2%)	93,053 (78.9%)	24,928 (21.1%)

	hoxd4a		*hoxd10a*		*hoxd11a*		*hoxd13a*	
	3′ signal	5′ signal	3′ signal	5′ signal	3′ signal	5′ signal	3′ signal	5′ signal
HoxDa	118,791 (74.4%)	40,811 (25.6%)	90,028 (63.9%)	50,941 (36.1%)	77,155 (55.1%)	62,809 (44.9%)	60,037 (34.5%)	114,230 (65.5%)
