# Supplementary material for: Conservation and Divergence of Regulatory Strategies at Hox Loci and the Origin of Tetrapod Digits
Source: PLoS Biol. 2014 Jan 21;12(1):e1001773. doi: 10.1371/journal.pbio.1001773 (PMC3897358; doi:10.1371/journal.pbio.1001773)
Supplement: Table S2 — List of primers used for 4C-seq analyses. (PDF) [file pbio.1001773.s007.pdf]

Table S2: 4C-seq primers

Mouse

	Name	Sequence
Hoxa4	*Hoxa4* iF*	AATGATACGGCGACCACCGAACACTCTTTCCCTACACGACGCTCTTCCGATCTAGCAAGGAGAGGAAACTAC
	Hoxa4 iR*	CAAGCAGAAGACGGCATACGAGTGGTGTTACTGTGCACTC
Hoxa9	*Hoxa9* iF*	AATGATACGGCGACCACCGAACACTCTTTCCCTACACGACGCTCTTCCGATCTAGGGATGCATAGATTCATG
	Hoxa9 iR*	CAAGCAGAAGACGGCATACGAACAGTGTTCAAGTATTTTGG
Hoxa11	*Hoxa11* iF	AATGATACGGCGACCACCGAACACTCTTTCCCTACACGACGCTCTTCCGATCTAGGGTGTACCTTGTGAGTCCTTTC
	Hoxa11 iR	CAAGCAGAAGACGGCATACGAGAGCTCAGGTTTGGGTGCAG
Hoxa13	*Hoxa13* iF*	AATGATACGGCGACCACCGAACACTCTTTCCCTACACGACGCTCTTCCGATCTAACACTTGCACAACCAGAAATGC
	Hoxa13 iR*	CAAGCAGAAGACGGCATACGAGGCGAGGCTCAGGCTTTTAT
Hoxd4	*Hoxd4* iF*	AATGATACGGCGACCACCGAACACTCTTTCCCTACACGACGCTCTTCCGATCTAAGGACAATAAAGCATCCATAGGCG
	Hoxd4 iR*	CAAGCAGAAGACGGCATACGATCCAGTGGAATTGGGTGGGAT
Hoxd11	*Hoxd11* iF*	AATGATACGGCGACCACCGAACACTCTTTCCCTACACGACGCTCTTCCGATCTAAGCATACTTCCTCAGAAGAGGCA
	Hoxd11 iR*	CAAGCAGAAGACGGCATACGACTAGGAAAATTCCTAATTTCAGG
Hoxd13	*Hoxd13* iF*	AATGATACGGCGACCACCGAACACTCTTTCCCTACACGACGCTCTTCCGATCTAAAAATCCTAGACCTGGTCATG
	Hoxd13 iR*	CAAGCAGAAGACGGCATACGAGGCCGATGGTGCTGTATAGG

* Primers from: Noordermeer D, Leleu M, Splinter E, Rougemont J, De Laat W, Duboule D (2011) The dynamic architecture of *Hox* gene clusters. Science 334: 222-225.

Zebrafish

	Name	Sequence
Hoxa4a	*Hoxa4a* iF	AATGATACGGCGACCACCGAACACTCTTTCCCTACACGACGCTCTTCCGATCTGAACAATGCATTGGTGAAAGG
	Hoxa4a iR	CAAGCAGAAGACGGCATACGAAACGAAAATAATCTTTGACTGCC
Hoxa9a	*Hoxa9a* iF	AATGATACGGCGACCACCGAACACTCTTTCCCTACACGACGCTCTTCCGATCTGCTAAGCCTATTTATAAAACACAGA
	Hoxa9a iR	CAAGCAGAAGACGGCATACGAAAATCTCTCGTTTTAACTCCAC
Hoxa11a	*Hoxa11a* iF	AATGATACGGCGACCACCGAACACTCTTTCCCTACACGACGCTCTTCCGATCTCCTTCATTGGGAGGCACTCGTGA
	Hoxa11a iR	CAAGCAGAAGACGGCATACGAGGGTAAAGTTGCCAGTTATTAG
Hoxa13a	*Hoxa13a* iF	AATGATACGGCGACCACCGAACACTCTTTCCCTACACGACGCTCTTCCGATCTTTGGACTACTGCTTTTGAAGTG
	Hoxa13a iR	CAAGCAGAAGACGGCATACGACGTACATCCAGAATCACGTC
Hoxa2b	*Hoxa2b* iF	AATGATACGGCGACCACCGAACACTCTTTCCCTACACGACGCTCTTCCGATCTTAGAAACGCAATATATTTGACAAG
	Hoxa2b iR	CAAGCAGAAGACGGCATACGAGACCAGGGTCGGTGGTTG
Hoxa11b	*Hoxa11b* iF	AATGATACGGCGACCACCGAACACTCTTTCCCTACACGACGCTCTTCCGATCTATGTTTTAGCAAAGATTTTAGATACC
	Hoxa11b iR	CAAGCAGAAGACGGCATACGACCACTTATATCAAACATTATCCG
Hoxa13b	*Hoxa13b* iF	AATGATACGGCGACCACCGAACACTCTTTCCCTACACGACGCTCTTCCGATCTTTCCCCAAGACTGAAAGAGAAG
	Hoxa13b iR	CAAGCAGAAGACGGCATACGATACTTTGGCTCCGAGTACAAC
Hoxd4a	*Hoxd4a* iF	AATGATACGGCGACCACCGAACACTCTTTCCCTACACGACGCTCTTCCGATCTAAAACAAGTCTCATACATCACAC
	Hoxd4a iR	CAAGCAGAAGACGGCATACGAATTTCTAAAGCGCTTTAGACAATG
Hoxd10a	*Hoxd10a* iF	AATGATACGGCGACCACCGAACACTCTTTCCCTACACGACGCTCTTCCGATCTCCTCAAACTGATACCTGGGCAG
	Hoxd10a iR	CAAGCAGAAGACGGCATACGACAGACGTGGTCAACAGCGACAC
Hoxd11a	*Hoxd11a* iF	AATGATACGGCGACCACCGAACACTCTTTCCCTACACGACGCTCTTCCGATCTATGCCCGTATTCCAAATACCAG
	Hoxd11a iR	CAAGCAGAAGACGGCATACGAACCTGAAACTAGCAACAGGCT
Hoxd13a	*Hoxd13a* iF	AATGATACGGCGACCACCGAACACTCTTTCCCTACACGACGCTCTTCCGATCTTTGACATGAATTTAAACAAATATGGA
	Hoxd13a iR	CAAGCAGAAGACGGCATACGATAAATTGATTATGTAATAACCAGGAG

Tetraodon

	Name	Sequence
Hoxa11b	*Hoxa11b* iF	AATGATACGGCGACCACCGAACACTCTTTCCCTACACGACGCTCTTCCGATCTCGTAGGCGGTGTCGAAGAAC
	Hoxa11b iR	CAAGCAGAAGACGGCATACGATCACCCACAGGAGAGGCTAAA
Hoxa13b	*Hoxa13b* iF	AATGATACGGCGACCACCGAACACTCTTTCCCTACACGACGCTCTTCCGATCTCGTATTAACTTAAACGATTATTG
	Hoxa13b iR	CAAGCAGAAGACGGCATACGATTAGTTGACCATTCTGTTTTC
Hoxd11a	*Hoxd11a* iF	AATGATACGGCGACCACCGAACACTCTTTCCCTACACGACGCTCTTCCGATCTACTCGTTCTTGAAAATGACATCC
	Hoxd11a iR	CAAGCAGAAGACGGCATACGAGCTGTTAAGGGTTATGTGCTTC
