# Supplementary material for: Conservation and Divergence of Regulatory Strategies at Hox Loci and the Origin of Tetrapod Digits
Source: PLoS Biol. 2014 Jan 21;12(1):e1001773. doi: 10.1371/journal.pbio.1001773 (PMC3897358; doi:10.1371/journal.pbio.1001773)
Supplement: Table S3 — List of primers used to clone DNA probe for whole mount in situ hybridizations. (PDF) [file pbio.1001773.s008.pdf]

Table S3: Primers used to clone ISH probes.

Probe	Primer sequences
mmu *Hoxa9*	CTGGGCAACTACTATGTGGAC
	GCTTGTCTCCGCCGCTCTCATTC
mmu *Hoxa11* as exon1	ATGATGGATTTTGATGAGCGTG
	TTAGAGAAGTGGATTAGCTGAG
tni *Hoxa11a*	ATGGATTTTGACGAACGGGTC
	TGCTGCTGCCGGATTTCTCCTC
tni *Hoxa13a*	ATGACAACGTCACTGCTCCTCC
	CTGCTACGGAAGACTTCCACAT
tni *Evx1*	TCTGGTTAGAGGCACTTCCTC
	GTCTGGTTTCAAAACCGCCG
tni *Hoxa10b*	ATGACATGCCCCAACAACC
	CTTTCACAGATTTCTCTGGACAT
tni *Hoxa11b*	ATGGATTTTGATGAGCGCGTC
	GCTTCTGCCCTGCTTCTCCTC
tni *Hoxa13b*	GTCATGTATAAATCTTTTCTTTTTAC
	TTTCTAAACAAACGTGCGAC
tni *HIBADHb*	GATCTGGGACTCGCTCAGAA
	TTCTGATTCCACTGTTTTATTTTG
tni *TAX1BP1b*	CATGGACATGAAGCGCTGTC
	TTTAAATCCAACTTTATTAGTTAATC
tni *JAZF1b*	GCAGCGAGTGCGACGAAGAGG
	CTGCTGCATCTTGCGGATG
tni *Hoxd4a*	ATGGCCATGAGTTCGTATATG
	TCAGTCTGGTCCTTGTGGATAC
tni *Hoxd9a*	ATGTCGACCAGTGGCACTC
	GTTTCTGCTCTTTGTGCTCGC
tni *Hoxd10a*	CTAACACGTTTTTAGTGGAGTC
	GGCTGGACGCCGACTCTTC
tni *Hoxd11a*	GCGCCTCCAGCATGTATCTG
	CTGCTGGTGGTGGTGGTTG
tni *Hoxd12a*	GTGCTCCTTGGTGCTCCTCAC
	GCTGTGCGAACACTCTTCTTC
tni *Evx2*	GTGTGGTTCCAGAACAGGAG
	GAACCCATCTCTGAAGCAG
